# Tailored Fano resonance and localized electromagnetic field enhancement in Ag gratings

**DOI:** 10.1038/srep44335

**Published:** 2017-03-14

**Authors:** Zhaozhu Li, J. Michael Klopf, Lei Wang, Kaida Yang, Rosa A. Lukaszew

**Affiliations:** 1 The College of William and Mary, Department of Physics, Williamsburg, VA, 23187, USA; 2 Helmholtz Zentrum Dresden-Rossendorf, Institute for Radiation Physics, Dresden, 01324, Germany; 3 University of Virginia, Dept. of Mechanical & Aerospace Engineering, Charlottesville, VA, 22904, USA

## Abstract

Metallic gratings can support Fano resonances when illuminated with EM radiation, and their characteristic reflectivity versus incident angle lineshape can be greatly affected by the surrounding dielectric environment and the grating geometry. By using conformal oblique incidence thin film deposition onto an optical grating substrate, it is possible to increase the grating amplitude due to shadowing effects, thereby enabling tailoring of the damping processes and electromagnetic field couplings of the Fano resonances, hence optimizing the associated localized electric field intensity. To investigate these effects we compare the optical reflectivity under resonance excitation in samples prepared by oblique angle deposition (OAD) and under normal deposition (ND) onto the same patterned surfaces. We observe that by applying OAD method, the sample exhibits a deeper and narrower reflectivity dip at resonance than that obtained under ND. This can be explained in terms of a lower damping of Fano resonance on obliquely deposited sample and leads to a stronger localized electric field. This approach opens a fabrication path for applications where tailoring the electromagnetic field induced by Fano resonance can improve the figure of merit of specific device characteristics, e.g. quantum efficiency (QE) in grating-based metallic photocathodes.

Fano resonances were first introduced theoretically by Ugo Fano to explain the phenomenon of auto-ionization of Helium atoms^1^. The Fano resonance occurs when the continuum or bright mode in an electromagnetic wave interferes constructively and destructively with a discrete dark mode. Experimentally the angle-dependent reflectivity of such resonance typically exhibits an asymmetric line-shape profile. This type of resonance has been observed in several systems such as coupled clusters of metallic nano-particles^2^,^3^,^4^, metallic photonic crystals^5^ and metamaterials^2^,^6^,^7^,^8^,^9^, etc. It is worth noting that this type of resonance can be used in applications including sensing^10^,^11^, optical modulators^12^,^13^,^14^, selective optical polarizers^15^, etc., because of its unique properties. However, classical optical phenomena such as the Wood anomaly resulting from the interference between Surface Plasmon Resonance (SPR) with radiative diffraction orders have only recently been clearly understood^2^,^16^.

We have investigated geometric effects on the Fano resonance excited on grating couplers due to its high sensitivity to the surrounding optical environment^17^,^18^. Several technologies for studying grating couplers have been investigated in recent times, including lithography methods^19^, self-assembly^20^,^21^, and physical vapor conformal deposition onto patterned substrates. Here we apply oblique angle deposition^22^ (OAD) to the Ag vapor flux using DC sputtering to form a film conformal to a patterned substrate. Such approach might offer advantages^23^ over traditional lithographic techniques for large-scale thin film fabrication.

We also investigated the enhancement of the electromagnetic field in such Ag-coated gratings and compare it with samples where the film has been deposited at normal Ag influx deposition (ND) onto identically patterned substrates. In order to correlate the optical response of such structures with the grating geometry, the surface morphology was probed using atomic force microscopy (AFM) in both cases, while the optical properties were characterized by experimental measurements of the reflectivity to detect the effect of the Fano resonance excitation on the SPR response. Electromagnetic simulations corresponding to the two types of grating structures have also been performed to better understand how the grating structure affects the localized electric field.

## Results

### Surface morphology and grating structure

**Figure 1:**
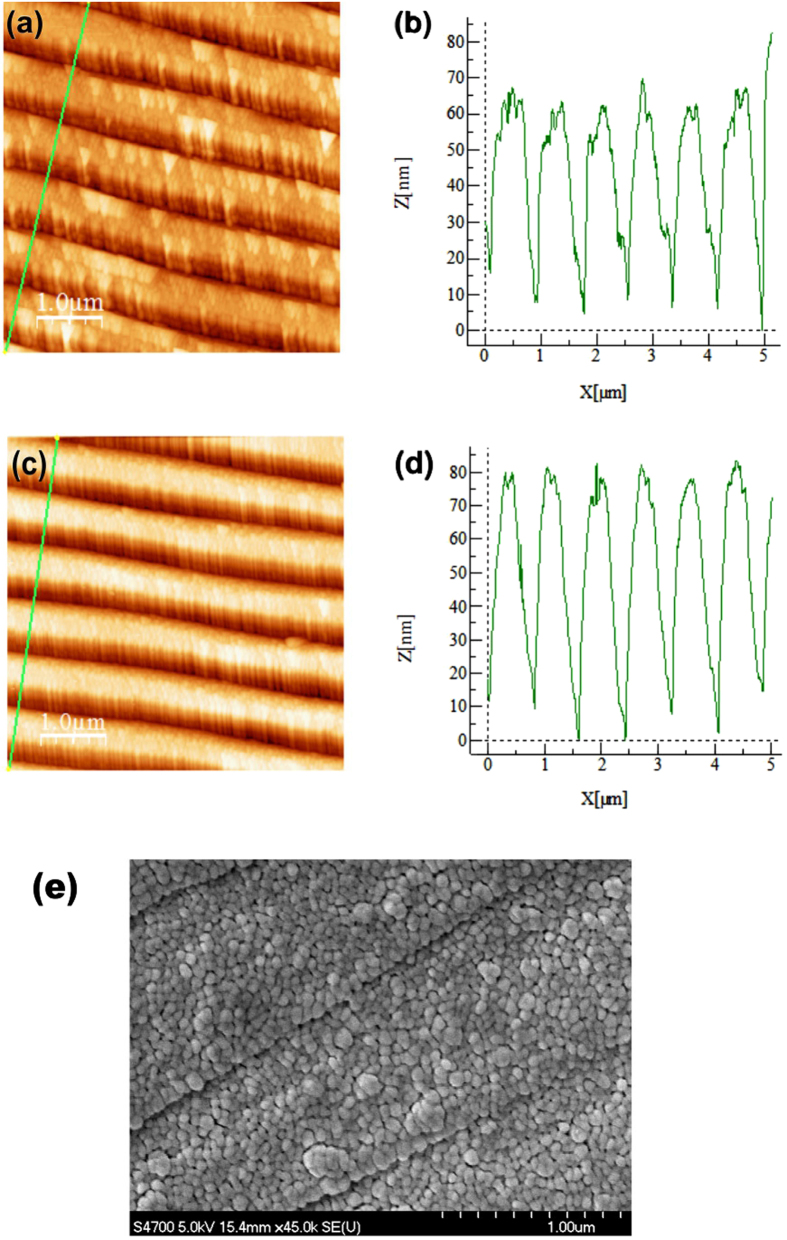
(**a**) Topography image (5 μm × 5 μm) for an ND Ag 59 nm grating sample. (**b**) Line scan along the path shown in green in (**a**). (**c**) Topography image (5 μm × 5 μm) for an OAD Ag 50 nm grating sample. (**d**) Line scan along the path shown in green in (**c**). (**e**) SEM image of OAD sample which shows full coverage of silver thin film.

The surface morphology of both types of samples was investigated by imaging the surfaces using atomic force microscopy (AFM), shown in Fig. 1. The AFM images show a clear difference in grating amplitude for these two different types of samples. For the OAD sample, the grating amplitude of the deposited Ag thin film (~80 nm) is 15 nm higher than that of the sample grown under normal incidence geometry (~65 nm). This difference is due to shadowing effects from the underlying pattern stemming from the large incident angle (*θ* ≈ 77°) of the Ag influx onto the grating substrate during deposition. Note here that because of the steepness of the trajectory of the AFM tip during scans over the grating surface, the groove depth values for both samples could have been experimentally under-estimated.

### Reflectivity measurements

**Figure 2:**
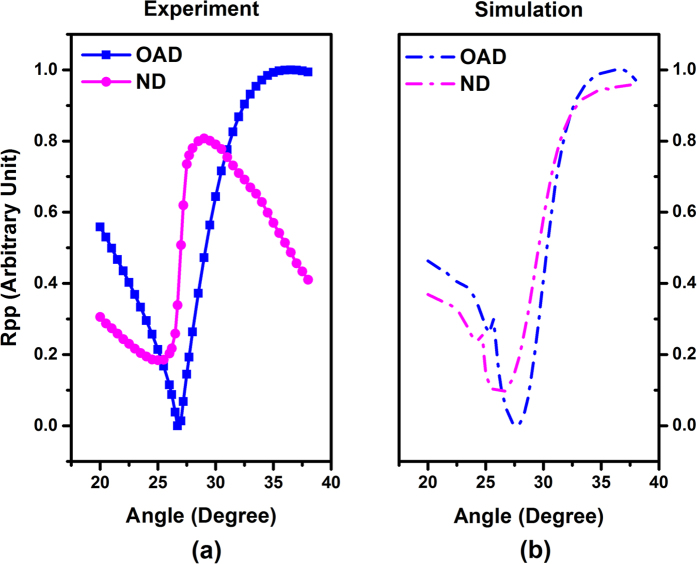
Reflectivity (Rpp) vs incidence angle (**a**) Experimental measurements and (**b**) simulations for OAD (blue) and ND (magenta) samples.

The p-polarized reflectivity versus incident light angle was experimentally investigated on both types of samples. The samples were illuminated with 405 nm p-polarized light, with the plane of incidence perpendicular to the direction of the grooves in the patterned surface. The incidence angle was varied between 20 and 38 degrees to obtain reflectivity vs incident-angle data. The experimental reflectivity results for typical OAD and ND samples are shown in Fig. 2(a). We observe a significant difference in the shape of the reflectivity curve at the SPR angle between these two samples. A narrower and sharper resonance is noticed for the sample grown using OAD compared to the ND one. We point out that the experimental results for both samples were normalized with same parameters such as the maximum and minimum reflectivity achieved with the OAD sample. We followed the same normalization method in the simulations. This normalization was carried out to aid the comparison between samples and between experimental results and simulations.

### Simulation results

**Figure 3:**
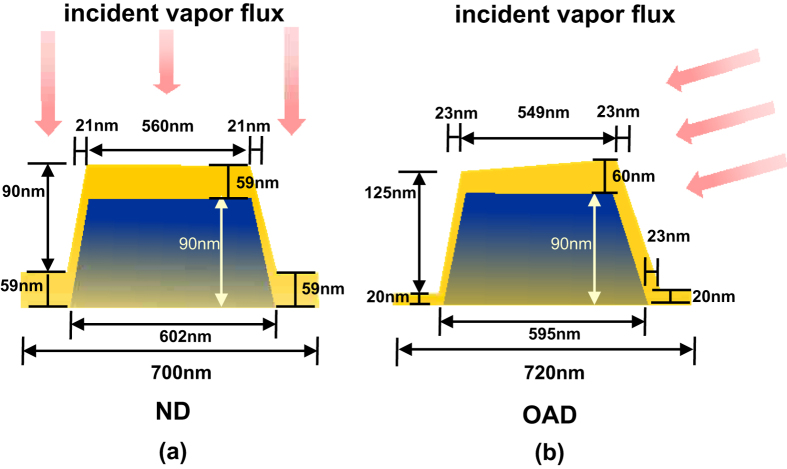
Illustration of the simulated grating profile: (**a**) ND with a nominal Ag thickness of 59 nm and (**b**) OAD with an uneven Ag deposition thickness from 20 to 60 nm.

We carried out simulations of both grating structures using the amplitude and pitch experimentally obtained under both thin film deposition geometries using EM Explorer software^24^. Using the dimensions obtained from the AFM line scan profile measurements across the surface topography of uncoated gratings, we simulated an isosceles trapezoidal profile using the same nominal dimensions for the substrate grating profile in both types of samples. The dimensions of the Ag film layer for each of the two simulated structures were set according to the AFM measurements for the OAD and ND samples. An illustration of the grating profile used in both cases is shown in Fig. 3. The reflectivity curves achieved with such simulations for both types of samples are shown in Fig. 2(b) and compared to the experimental results in Fig. 2(a). For the simulations, the Ag thin film follows the trapezoidal structure of the grating substrate homogeneously in the ND sample, while the Ag thin film in the OAD sample was distributed asymmetrically on either side of the stripe to accommodate for the shadowing effects of the oblique depostion^25^. The average thickness of the Ag thin films was set at ~60 nm for the simulated structures, agreeing with the measured profiles in the real samples. The geometrical distribution of the Ag film used to simulate the ND and OAD samples are indicated in Fig. 3(a) and (b), respectively. Note that the grating period used to better fit the ND sample results has been set at 700 nm while for the OAD, the grating pitch is set at 720 nm, an acceptable difference that is within the 5% variability of our commercial grating substrates (standard periodicity 740 nm). The simulated Ag thin film structures therefore accurately represent the two types of samples and account for the different deposition geometries investigated. The refractive index used for the simulation of the ND sample was n = 0.17, k = 2.10, while for the OAD sample n = 0.17, k = 2.20, was used. We kept the optical constants very close to each other in the simulations. In this way, we are able to examine the effect of a changing grating profile on the resonance, which is expected to have a more important role since we kept the Ag thickness similar on both samples. We note though, that the measured Ag thickness is slightly different between the two samples. This can be attributed to a different growth mode, e.g. different incident angles affecting growth rates, but we do not expect this to be a substantial effect. The value of the index of refraction used for the substrate was n = 1.56 for the simulations of both samples. Detailed information for both grating models can be observed in Fig. 3. Note that the very slight difference in the grating substrate dimensions for the OAD and ND models (e.g. 7 nm difference in the bottom width) is due to the slightly different resolution used in the simulations but this doesn’t affect the results. All the values of dielectric constants are within reasonable margins (<5%) from those obtained from ref. 26.

We also want to point out that the simulation model used for the OAD sample is justified because the actual profile of the grating substrate should allow full coverage of the Ag thin film coating even if not uniform., This is expected because reflections of incident atoms from the adjacent grating groove can also occur, allowing coating of the shadowed side of the groove considered. This phenomenon was observed in ref. 22 where the authors referred to it as “excessive deposition”. This full coverage of the groove is shown in Figs 1(e) and 3(b).

To understand the physical mechanisms governing our experimental optical observations regarding the difference between OAD and ND samples, we consider the process of a Fano resonance in a grating coupler. In the Fano resonance, a bright mode which is a radiative mode excited by the far-field illumination interferes with a dark mode or a localized mode. The total response is often found to be asymmetric around the resonance as it is shown in both the OAD and ND reflectivity curves (Fig. 2(a)). The main limitation of the original Fano resonance expression is that it lacks accounting of intrinsic losses due to the dark mode. Here we adopt Benjamin Gallinet and Olivier J. F. Martin’s description of the Fano resonance^27^,^28^,^29^, including consideration of intrinsic damping as shown in Eq. (1). This treatment enables understanding the influence of electromagnetic interactions on the resonance through the information drawn from a Fano profile.

The line profile for the dark mode is described in Eq. (1).


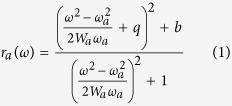


Here, *ω* is the angular frequency of the incident light, *ω*_*a*_ is the resonance central spectral frequency, *W*_*a*_ is the approximation of spectral width where *W*_*a*_ ≪ *ω*_*a*_, *b* represents the damping of the Fano resonance and affects the contrast of the resonance profile and *q* characterizes the asymmetry of the resonance profile.

**Figure 4:**
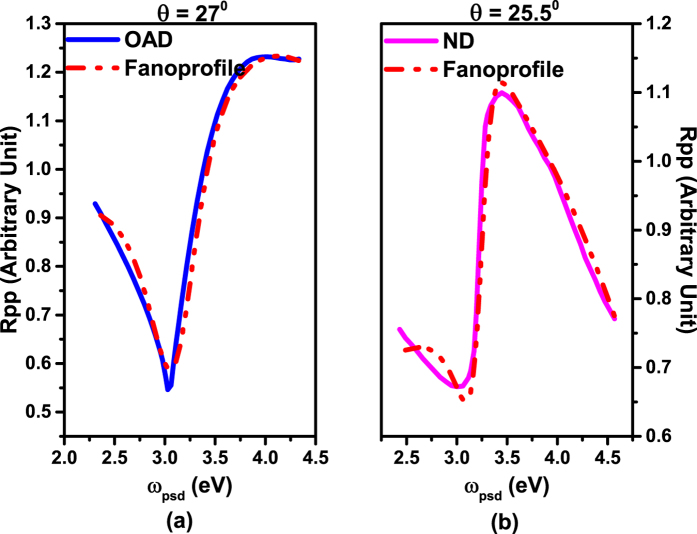
Fitting results of Fano resonance for reflectivity data for ND (**a**) Blue: Rpp data and red: fitting curve and OAD (**b**) magenta: Rpp data and red: fitting curve.

**Figure 5:**
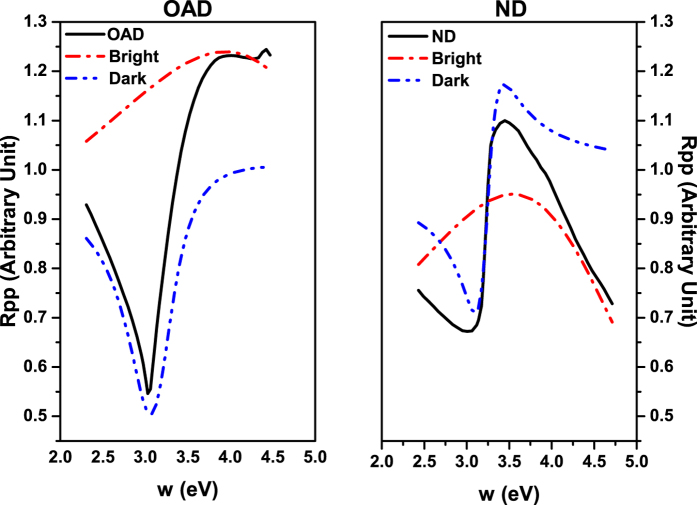
Fitted bright modes and dark modes for OAD (left) and ND (right) samples.

The bright mode can be constructed by pseudo Lorentzian form as in Eq. (2)


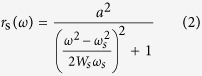


where *a* is the maximum amplitude, *ω*_*s*_ is the resonance frequency, and *W*_*s*_ is the approximation spectral width if *W*_*s*_ ≪ *ω*_*s*_. The total response of the system is Eq. (3), given by the product of Eqs (1) and (2), and can be applied in measurable quantities for fitting parameters.


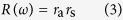


when light shines on a grating coupler, its horizontal component of momentum can be expressed as


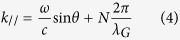


where *θ* is the incident angle, *λ*_*G*_ is the grating periodicity, *N* is the diffraction order, and *c* is the speed of light. We measured the 0^th^ order reflectivity, and the SPR appears when the 1^st^ diffraction order vanishes. Thus we set *N* as 1 and grating periodicity as the standard value *λ*_*G*_ = 740 nm. The reflectivity response of the incident angle in Fig. 2(a) is caused by the response of *k*_//_ on incident angle. Thus we first calculate the value of *k*_//_ for each *θ* of 405 nm light by Eq. 4. The reflectivity can now be expressed as a function of *k*_//_. To better analyze the physics mechanism of this Fano resonance, we propose a pseudo frequency *ω*_psd_ and a pseudo incident angle *θ*_psd_, and use *ω*_psd_ to represent the effect of incident angle on the *k*_//_ after setting a *θ*_psd_. We calculate (in Eq. 4) the value of a pseudo frequency *ω*_psd_ under each *k*_//_. In this way we convert the reflectivity data to a function of *ω*_psd_. One can plot the reflectivity versus pseudo-angular frequency *ω*_psd_ as in Fig. 4 (solid lines). The new reflectivity curves are then fitted by Eq. 3 using OriginLab^30^. We show the fitting parameters along with the standard errors in Table 1 and the fitting curves dashed in Fig. 4 (red). Note that we set incident angles for both samples at their respective resonance angles. We find that varying the pseudo incident angle from 20 to 38 degrees has very little effect on the result of *q* and *b* for both types of samples, evidencing a characteristic relationship between these two parameters and the Fano profile itself within the considered angle range. The dark mode and bright mode for each sample are plotted as in Fig. 5 as an example. Note that the term including grating periodicity is cancelled during the calculations for each sample and the only changing part in eq. (4) is the term with *θ* thus it doesn’t affect the fitting results.

We see that, in our fitting results, the asymmetry parameter *q* for the OAD samples is much smaller than for the ND samples and its reflectivity shows a near symmetric resonance profile. On the other hand, the value of the modulation damping parameter *b* for the OAD samples is noticeably lower than that of the ND samples with good accuracy. The imaginary part of the dielectric permittivity in a metal would be accountable for the damping effect when there is interference between bright and dark modes, and it is considered an intrinsic loss. This damped interference displays either incomplete construction or destruction effects on the Fano profile, and the *b* parameter evaluates such effect by comparing the ratio between the intensity lost to the metallic structure and the intensity transferred from the radiative mode to a localized mode. When *b* equals 0, the intrinsic losses would fully disappear and the reflectivity should reach 0 at the resonance angle. A lower value of the *b* parameter indicates lower ratio of intrinsic losses coupling radiative and localized modes. In our case, with the consideration of similar dielectric permittivity, a stronger coupling between radiative modes and localized modes in the OAD samples should be the cause of lower damping compared to the ND samples. Thus, we can conclude that the main enhancement effect in the resonance is due to lower damping in the samples grown by OAD than that of the ND ones. Such scenario suggests the existence of an associated enhanced electromagnetic field in the OAD samples, which could benefit many applications, such as photoemission from metallic photocathodes, etc. We believe that the bright mode in the Fano resonance corresponds to the 1st order diffraction in the grating as discussed in ref. 16. After diffraction of the incident of the light, the 1st order diffraction is generated and couples to the surface plasmon resonance, which is a localized mode in the Ag grating. The first order diffraction generated also diffracts and contributes to the homogenous zeroth order. As a result, the zero order diffraction is the superposition of the two interactions mentioned above.

**Figure 6:**
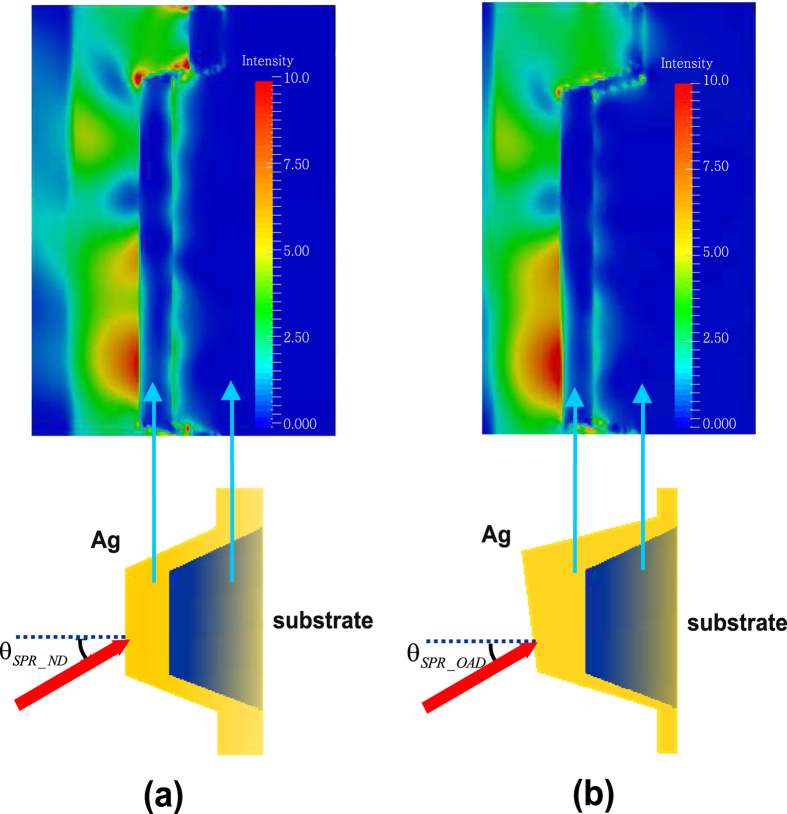
Electric field intensity of both samples at its resonance angle. (**a**) ND Ag sample, nominal deposition thickness of Ag 59 nm. (**b**) OAD Ag sample, nominal deposition thickness of Ag 50 nm. All intensities that are higher than 10 are set as red.

To examine this effect in both the OAD and ND samples, we also simulated the electromagnetic field profile based on the previous model described. These simulations were carried out under the same illumination conditions as in our experiments, i.e., at the same wavelength, fluence, and critical angle as experimentally determined. The magnitude of the electric fields computed by these simulations is shown in Fig. 6. The expected stronger EM fields near the surface of the deeper-grooved (OAD) sample compared to the shallower grooved sample (ND) is confirmed, and it is attributed to a lower damping of the Fano resonance which indicates a stronger coupling of the first diffraction order with the SPR excited on the grating structure^16^. Thus we can conclude that the OAD method offers a viable approach to tailor local electromagnetic field enhancements near the surface in grating-induced Fano resonances due to our ability to control damping processes.

## Conclusion

In conclusion, we have applied conformal thin film growth techniques using different influx geometries during film deposition to tailor Ag thin film gratings using DC sputtering onto commercial optical gratings. We have compared the experimental surface topography and reflectivity curves for samples fabricated under OAD and ND geometries. Analysis of the topography reveals a somewhat deeper groove pattern for the sample obtained by OAD compared to the one obtained by ND. The enhanced optical response of the OAD sample is demonstrated by a deeper and narrower dip in the measured reflectance at the resonance angle when the Fano resonance is excited and by the higher localized electric field computed in the simulations. This enhanced Fano resonance mode is attributed to lower damping caused from stronger coupling of bright and dark modes in Ag thin film grating structures in OAD samples. These results demonstrate the possibility of enhancing the quantum efficiency in devices such as metallic photocathodes by tailoring the surface profile and hence the associated damping mechanisms at play when using plasmonic approaches such as the ones described here.

## Methods

### OAD method and sample preparation

**Figure 7:**
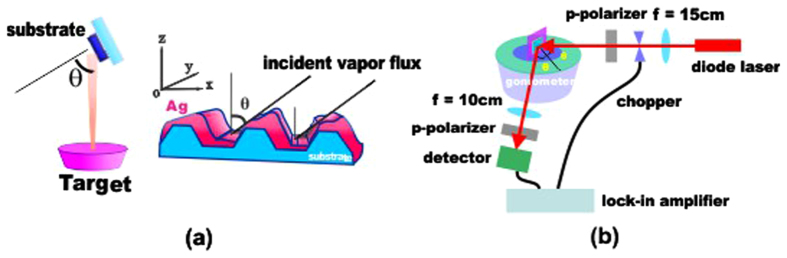
(**a**) Illustration of OAD method and its shadowing effects of sputtering deposition on gratings. (**b**) Experimental setup to measure SPR on Ag grating samples.

An illustration of OAD deposition is shown in Fig. 7(a). The sputtered beam flux is incident at angle *θ* ≈ 77° relative to the normal direction of the grooved substrate. Because of shadowing effects from the underlying grating groove (Fig. 7(a)), asymmetric accumulation of impingent flux on the grating allows for tailoring of the resulting structure as indeed we observed. The substrates for both OAD and ND samples were loaded into a vacuum chamber with base pressure at 2.0 E-6 torr and the Ag films were grown under an Ar pressure of 7.5 E-3 torr using DC magnetron sputtering, from the same Ag target (99.9% pure) onto commercial patterned substrates, i.e., polycarbonate diffraction optical gratings. The Ag film deposition was carried out to achieve similar thickness films for both samples – around 60 nm since this thickness region was previously demonstrated to follow conformal growth. The nominal periodicity of the grating substrate is 740 nm with a 5% tolerance.

### Experiment setup for SPR measurement

The OAD and ND structure samples were mounted on a rotational stage in a custom-built high-precision goniometer system. Each sample was mounted such that the grating grooves were normal to the plane of light incidence. A p-polarized blue diode laser (*λ* = 405 nm) was modulated with a 255 Hz optical chopper mounted in the path of the illuminating incident beam, and the incidence angle was varied from 20° to 38° in 0.5° increments. We used a Si photodetector (Thorlabs DET10A) and a lock-in amplifier from Standard Research Systems (SR510) to measure the zero-order reflected beam. The sample surface was aligned to the center of rotation to ensure optimal measurements in the same region of the sample surface at all angles. A schematic illustration of the optical SPR setup is shown in Fig. 7(b).

## Additional Information

**How to cite this article:** Li, Z. *et al*. Tailored Fano resonance and localized electromagnetic field enhancement in Ag gratings. *Sci. Rep.* **7**, 44335; doi: 10.1038/srep44335 (2017).

**Publisher's note:** Springer Nature remains neutral with regard to jurisdictional claims in published maps and institutional affiliations.

## Figures and Tables

**Table 1:** Fitting Parameters of Fano Resonance Profile of Eq. (3), both incident angles of OAD and ND sample at its SPR angle, respectively.

	OAD		ND	
	Fitted Value	Standard Error	Fitted Value	Standard Error
*w*_*a*_	3.10575	0.01979	3.22967	0.00828
*w*_*s*_	3.94352	0.10329	3.53975	0.03437
*W*_*a*_	0.32953	0.01436	0.16508	0.00885
*W*_*s*_	3.12978	0.52405	2.22655	0.11962
*q*	0.06475	0.02458	0.22555	0.0093
*b*	0.5083	0.00914	0.83131	0.02203
*a*	1.11337	0.00989	0.97496	0.00477

Standard errors are defined as in OriginLab Non-linear Fitting.
